# The Global Landscape of Pediatric Bacterial Meningitis Data Reported to the World Health Organization–Coordinated Invasive Bacterial Vaccine-Preventable Disease Surveillance Network, 2014–2019

**DOI:** 10.1093/infdis/jiab217

**Published:** 2021-09-01

**Authors:** Tomoka Nakamura, Adam L Cohen, Stephanie Schwartz, Jason M Mwenda, Goitom Weldegebriel, Joseph N M Biey, Reggis Katsande, Amany Ghoniem, Kamal Fahmy, Hossam Abdel Rahman, Dovile Videbaek, Danni Daniels, Simarjit Singh, Annemarie Wasley, Gloria Rey-Benito, Lucia de Oliveira, Claudia Ortiz, Emmanuel Tondo, Jayantha B L Liyanage, Mohammad Sharifuzzaman, Varja Grabovac, Nyambat Batmunkh, Josephine Logronio, James Heffelfinger, Kimberly Fox, Linda De Gouveia, Anne von Gottberg, Mignon Du Plessis, Brenda Kwambana-Adams, Martin Antonio, Samaa El Gohary, Aya Azmy, Asmaa Gamal, Elena Voropaeva, Ekaterina Egorova, Yulia Urban, Carolina Duarte, Balaji Veeraraghavan, Samir Saha, Ben Howden, Michelle Sait, Sangoun Jung, Songmee Bae, David Litt, Shila Seaton, Mary Slack, Sebastien Antoni, Mahamoudou Ouattara, Chris Van Beneden, Fatima Serhan

**Affiliations:** 1 Department of Immunization, Vaccines and Biologicals, World Health Organization, Geneva, Switzerland; 2 Division of Bacterial Diseases, US Centers for Disease Control and Prevention, Global Reference Laboratory for the WHO-coordinated Invasive Bacterial Vaccine Preventable Disease Surveillance Network, National Center for Immunization and Respiratory Disease, Atlanta, Georgia, USA; 3 Department of Vaccine Preventable Diseases Program, World Health Organization Regional Office for Africa, Brazzaville, Congo Republic; 4 Department of Immunization, Vaccines and Biologicals, World Health Organization Regional Office for Africa, Inter-Support Team for East and South Africa, Harare, Zimbabwe; 5 Department of Vaccine Preventable Diseases, World Health Organization Regional Office for Africa, Inter-Support Team for West Africa, Ouagadougou, Burkina Faso; 6 Department of Communicable Diseases, Immunization, Vaccines and Biologicals Unit, World Health Organization Eastern Mediterranean Office, Cairo, Egypt; 7 Division of Country Health Programmes, Vaccine-Preventable Diseases and Immunization Unit, World Health Organization European Regional Office, Copenhagen, Denmark; 8 Pan American Health Organization/Department of Family, Health Promotion, and Life Course, World Health Organization Regional Office for the Americas, Comprehensive Family Immunization Unit, Washington DC, USA; 9 Department of Immunization and Vaccine Development, World Health Organization South-East Asia Regional Office, New Delhi, India; 10 Division of Programmes for Diseases Control, Vaccine Preventable Diseases and Immunization, World Health Organization Western Pacific Regional Office, Manila, Philippines; 11 Division of the National Health Laboratory Service, National Institute for Communicable Diseases, African Regional Reference Laboratory For The WHO-coordinated Invasive Bacterial Vaccine Preventable Disease Surveillance Network, Centre for Respiratory Diseases and Meningitis, Johannesburg, South Africa; 12 University of the Witwatersrand, School of Pathology, Faculty of Health Sciences, Johannesburg, South Africa; 13 Medical Research Council Unit The Gambia at the London School of Hygiene and Tropical Medicine, WHO Collaborating Centre for New Vaccines Surveillance and African Regional Reference Laboratory for the WHO-coordinated Invasive Bacterial Vaccine Preventable Disease Surveillance Network, Fajara, Banjul, The Gambia; 14 Department of Clinical Bacteriology Development, Central Public Health Laboratories, Eastern Mediterranean Region Regional Reference Laboratory for the WHO-coordinated Invasive Bacterial Vaccine Preventable Disease Surveillance Network, Cairo, Egypt; 15 G.N. Gabrichevsky Research Institute for Epidemiology and Microbiology, Laboratory of Clinical Microbiology and Biotechnology, European Regional Reference Laboratory for the WHO-coordinated Invasive Bacterial Vaccine Preventable Disease Surveillance Network, Moscow, Russian Federation; 16 Instituto Nacional de Salud, Dirección de Redes en Salud Pública, Regional Reference Laboratory for the WHO-coordinated Invasive Bacterial Vaccine Preventable Disease Surveillance Network, Bogotá, D.C., Colombia; 17 Department of Clinical Microbiology, Christian Medical College and Hospital, South-East Asia Regional Reference Laboratory for the WHO-coordinated Invasive Bacterial Vaccine Preventable Disease Surveillance Network, Vellore, Tamil Nadu, India; 18 Department of Microbiology, Bangladesh Institute of Child Health and Child Health Research Foundation, South-East Asia Region National Laboratory for the WHO-coordinated Invasive Bacterial Vaccine Preventable Disease Surveillance Network, Dhaka, Bangladesh; 19 The Peter Doherty Institute for Infection and Immunity, Microbiological Diagnostic Unit Public Health Laboratory, Western Pacific Region Regional Reference Laboratory for the WHO-coordinated Invasive Bacterial Vaccine Preventable Disease Surveillance Network, Melbourne, Australia; 20 Division of Bacterial Disease, Korea Disease Control and Prevention Agency, Western Pacific Region Regional Reference Laboratory for the WHO-coordinated Invasive Bacterial Vaccine Preventable Disease Surveillance Network, Cheongju-Si, Chungcheongbuk-do, Republic of Korea; 21 Division of Tuberculosis and Bacterial Respiratory Infections, Korea Disease Control and Prevention Agency, Western Pacific Region Regional Reference Laboratory for the WHO-coordinated Invasive Bacterial Vaccine Preventable Disease Surveillance Network, Cheongju-Si, Chungcheongbuk-do, Republic of Korea; 22 Public Health England, Respiratory and Vaccine Preventable Bacteria Reference Unit, WHO Collaborating Center for Haemophilius and Streptococcus pneumoniae, London, United Kingdom; 23 Public Health England, United Kingdom National External Quality Assessment Services, London, United Kingdom

**Keywords:** invasive bacterial disease, surveillance, vaccine preventable disease, meningitis, pneumococcal, meningococcal, pneumococcal conjugate vaccine

## Abstract

**Background:**

The World Health Organization (WHO) coordinates the Global Invasive Bacterial Vaccine-Preventable Diseases (IB-VPD) Surveillance Network to support vaccine introduction decisions and use. The network was established to strengthen surveillance and laboratory confirmation of meningitis caused by *Streptococcus pneumoniae*, *Haemophilus influenzae*, and *Neisseria meningitidis*.

**Methods:**

Sentinel hospitals report cases of children <5 years of age hospitalized for suspected meningitis. Laboratories report confirmatory testing results and strain characterization tested by polymerase chain reaction. In 2019, the network included 123 laboratories that follow validated, standardized testing and reporting strategies.

**Results:**

From 2014 through 2019, >137 000 suspected meningitis cases were reported by 58 participating countries, with 44.6% (n = 61 386) reported from countries in the WHO African Region. More than half (56.6%, n = 77 873) were among children <1 year of age, and 4.0% (n = 4010) died among those with reported disease outcome. Among suspected meningitis cases, 8.6% (n = 11 798) were classified as probable bacterial meningitis. One of 3 bacterial pathogens was identified in 30.3% (n = 3576) of these cases, namely *S. pneumoniae* (n = 2177 [60.9%]), *H. influenzae* (n = 633 [17.7%]), and *N. meningitidis* (n = 766 [21.4%]). Among confirmed bacterial meningitis cases with outcome reported, 11.0% died; case fatality ratio varied by pathogen (*S. pneumoniae*, 12.2%; *H. influenzae*, 6.1%; *N. meningitidis*, 11.0%). Among the 277 children who died with confirmed bacterial meningitis, 189 (68.2%) had confirmed *S. pneumoniae*. The proportion of pneumococcal cases with pneumococcal conjugate vaccine (PCV) serotypes decreased as the number of countries implementing PCV increased, from 77.8% (n = 273) to 47.5% (n = 248). Of 397 *H. influenzae* specimens serotyped, 49.1% (n = 195) were type b. Predominant *N. meningitidis* serogroups varied by region.

**Conclusions:**

This multitier, global surveillance network has supported countries in detecting and serotyping the 3 principal invasive bacterial pathogens that cause pediatric meningitis. *Streptococcus pneumoniae* was the most common bacterial pathogen detected globally despite the growing number of countries that have nationally introduced PCV. The large proportions of deaths due to *S. pneumoniae* reflect the high proportion of meningitis cases caused by this pathogen. This global network demonstrated a strong correlation between PCV introduction status and reduction in the proportion of pneumococcal meningitis infections caused by vaccine serotypes. Maintaining case-based, active surveillance with laboratory confirmation for prioritized vaccine-preventable diseases remains a critical component of the global agenda in public health.

The World Health Organization (WHO)-coordinated Invasive Bacterial Vaccine-Preventable Disease (IB-VPD) Surveillance Network reported data from 2014 to 2019, contributing to the estimates of the disease burden and serotypes of pediatric meningitis caused by Streptococcus pneumoniae, Haemophilus influenzae and Neisseria meningitidis.

*Streptococcus pneumoniae* (Sp), *Haemophilus influenzae* (Hi), and *Neisseria meningitidis* (Nm) are the major causes of bacterial meningitis in children <5 years of age worldwide, particularly in countries with limited resources [1]. Extensive use of Hi type b (Hib) and pneumococcal conjugate vaccines (PCVs) have resulted in marked decline in the global disease burden of Hib and pneumococcal diseases [2]. The introduction of MenAfriVac, a meningococcal group A conjugate vaccine rolled out in the African meningitis belt countries, has also led to a decline in the incidence of suspected meningitis and epidemic risk [3].

The World Health Organization (WHO) has overseen and coordinated the Global Invasive Bacterial Vaccine-Preventable Disease (IB-VPD) Surveillance Network (GISN) since 2009. The network was established to standardize the monitoring of the global burden and etiology of IB-VPD and to support PCV introduction and monitor its impact, primarily in low- and middle-income countries (LMICs) with limited surveillance and laboratory capacity [4, 5]. The laboratory network of GISN was developed as a multitier structure of global, regional, and national laboratories to provide technical assistance and guidance to support country-level surveillance activities in meningitis and other invasive bacterial diseases. The network followed operational modalities similar to other WHO-coordinated laboratory networks for vaccine-preventable diseases with tiered-approach technical support from global and regional laboratories to national and sentinel site laboratories [6].

Surveillance of vaccine-preventable diseases can provide critical information for policymakers; data on Sp, Hi, and Nm in LMICs are limited [7]. Robust surveillance data also aid the early detection and management of meningococcal or pneumococcal outbreaks [8]. The aim of this report is to describe the etiology, serotype and serogroup, epidemiology, and clinical presentations of pediatric bacterial meningitis in LMICs contributing to this global network.

## METHODS

### Case Definitions, Enrollment, and Data Collection

Between 2014 and 2019, active, case-based meningitis surveillance was conducted by 149 participating sentinel hospital surveillance sites in 58 WHO Member States from all 6 WHO regions: the African Region, Region of the Americas, Eastern Mediterranean Region, European Region, South-East Asia Region, and Western Pacific Region.

Sentinel site hospitals conducting meningitis surveillance through GISN are asked to report all suspected meningitis infections in hospitalized children aged <5 years to WHO on a monthly basis. WHO headquarters receive the compiled surveillance data quarterly from each WHO regional office. Suspected meningitis is defined as illness in a child aged 0–59 months admitted to a hospital with sudden-onset fever (>38.5°C rectal or 38°C axillary) and 1 of the following signs: neck stiffness, altered consciousness with no other alternative diagnosis, or other meningeal signs; or illness in any patient aged 0–59 months who is hospitalized with a clinical diagnosis of meningitis [9]. The sentinel site hospitals are asked to collect cerebrospinal fluid (CSF) from all children with suspected meningitis. Results of laboratory diagnostics are then used to further classify suspected meningitis cases. Standardized case definitions for suspected, probable, and confirmed bacterial meningitis (Table 1) are used by all participating hospitals [9].

**Table 1.** Meningitis Case Definitions

Suspected meningitis	Illness in a child aged 0–59 mo admitted to hospital with sudden-onset fever (>38.5°C rectal or 38°C axillary) and 1 of the following signs: neck stiffness, altered consciousness with no other alternative diagnosis, or other meningeal signs; or illness in any patient aged 0–59 mo who is hospitalized with a clinical diagnosis of meningitis.
Probable bacterial meningitis	A suspected meningitis case with CSF examination showing at least 1 of the following: turbid appearance; leukocytosis (>100 cells/μL); leukocytosis (10–100 cells/μL) AND either an elevated protein (>100 mg/dL) or decreased glucose (<40 mg/dL). If protein and glucose results are not available, diagnose using the first 2 conditions (turbid appearance or leukocytosis >100 cells/μL).
Confirmed bacterial meningitis	A suspected meningitis case or probable meningitis case that is laboratory-confirmed by growing (ie, culturing) or identification (ie, by Gram stain, antigen detection by immunochromatography or latex agglutination, polymerase chain reaction) of a bacterial pathogen (Hib, pneumococcus, or meningococcus) in the CSF or from the blood in a child with a clinical syndrome consistent with bacterial meningitis.

Abbreviations: CSF, cerebrospinal fluid; Hib, *Haemophilus influenzae* type b.

### Laboratory Methods

The initial laboratory analysis of CSF specimens collected from suspected meningitis cases include microscopy, culture, and rapid diagnostic tests (BinaxNOW for Sp detection and Pastorex Meningitis antigen rapid latex agglutination for Sp, Nm, and Hi) [10]. Cell count, glucose, and protein concentration from CSF samples are used to determine whether the cases are probable bacterial meningitis cases (Table 1). All sentinel site laboratories (SSLs) performed culture and could perform rapid diagnostic tests (RDTs); however, occasional disruptions to the supply chain for reagents occurred, and not all samples were tested by all methods.

Clinical specimens from suspected meningitis cases with sufficient residual material are referred to regional reference laboratories (RRLs) for molecular testing to identify Sp, Hi, or Nm and serotyping/serogrouping. In addition, all bacterial isolates are referred to RRLs for confirmation and molecular characterization. The PCR methods performed at all 9 RRLs were mainly adapted from the Global Reference Laboratory (GRL) based at the US Centers for Disease Control and Prevention (Atlanta, Georgia), including conventional and real-time PCR assays for the target *lytA* (Sp), *hpd* (Hi), and *SodC* or *ctrA* (Nm) genes for identification of the pathogens and multiplex PCR assays for serotyping/serogrouping [11–15]. In most laboratories, Sp, Hi, and Nm serotype/serogroup data reported were determined using PCR on CSF samples. All RRLs were testing the referred samples by PCR for detection of the 3 pathogens from CSF samples and for the serotyping/serogrouping of positive Sp, Hi, and Nm cases from CSF or bacterial isolates received. A limited number of RRLs in the network used the Quellung reaction on Sp isolates for determining the serotype.

**Figure 1.**
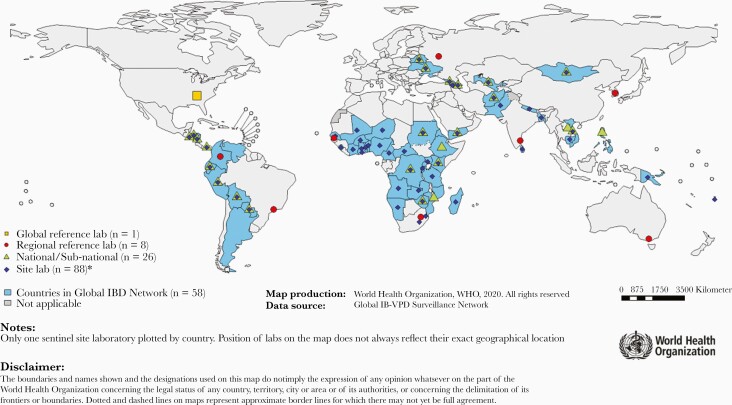
World Health Organization (WHO) Member States and network laboratories that reported surveillance and laboratory data to the WHO Global Invasive Bacterial Vaccine-Preventable Disease (IB-VPD) Surveillance Network, 2019.

As of 2019, the laboratory network of GISN included 88 sentinel site laboratories, 26 national laboratories (NLs), 8 RRLs, and 1 GRL (Figure 1). All participating laboratories in GISN follow validated, standardized testing and reporting strategies and undertake annual quality assurance and quality control exercises that are coordinated by WHO and technical partners. WHO recommends the freezing and storage of CSF from –20°C or –70°C at the SSLs or hospitals prior to sending to RRLs. When further characterization is necessary, RRLs can refer the specimens to the GRL.

### Quality Assurance

WHO has implemented global quality assurance programs that help assess the performance of laboratories. An annual external quality assessment (EQA) exercise is conducted in collaboration with the United Kingdom National External Quality Assessment Unit at Public Health England and consists of sending blinded proficiency testing panels of bacterial meningitis to the network laboratories. SSLs and NLs receive proficiency testing panels of bacterial cultures and Gram stain slides while RRLs and those SSLs and NLs having molecular testing capacities additionally receive CSF-simulated samples for molecular diagnosis and serotyping/serogrouping. A detailed report of the EQA results was shared annually with individual laboratories and helped identify areas for further technical support from RRLs or WHO.

Other measures of quality control were implemented at the regional level, such as a referral of randomly selected specimens and isolates collected from the sentinel or hospital laboratories to the corresponding RRLs for confirmatory testing, though this was not consistent in all regions and depended on available capacities and resources. At the global level, a standard external quality control exercise is conducted annually between the RRLs and GRL and consists of referring 50 previously characterized and select specimens (including a sampling of some that tested negative and positive) and isolates, from each RRL to the GRL for confirmatory testing. Discordant results are investigated and addressed to identify and remediate any issues or concerns that may impact the consensus of results. The results are also used to highlight areas of strength in capacity as well as to understand where additional training and resources should be focused for improvement.

### Data Analysis

This report analyzes cases reported from 2014 to 2019; prior to 2014, most of the WHO regions collected aggregate data rather than case-based data. Data management and statistical analysis were performed using Stata (version 16) and R (version 4.0.2) softwares. Statistical tests included the Pearson χ ^2^ test and the χ ^2^ test for trend to compare proportions of serotypes and serogroups over time and to assess the level of association between vaccine introduction status and whether the serotype/serogroup was a vaccine type or not.

For the analysis of Sp serotype distribution, vaccine types and nonvaccine types were categorized based on the type of PCV that was used as part of the country’s routine immunization program during a specific year. The 7-valent PCV (PCV7) includes serotypes 4, 6B, 9V, 14, 18C, 19F, and 23F; 10-valent PCV (PCV10) includes serotypes 1, 5, and 7F in addition to those covered in PCV7; 13-valent PVC (PCV13) includes 3, 6A, and 19A in addition to those covered in PCV13. If countries switched the PCV type used between 2014 and 2019, this was taken into account in the analysis. Although only serotypes 6A and 6B are covered by PCV13, serotypes 6C and 6D were combined together when analyzing the Sp serotype distribution due to the fact that while some laboratories use PCR (ie, can distinguish between 6A/6B and 6C/6D), some use Quellung as their routine procedure in serotyping (ie, can distinguish between all 4 serotypes). Pre–PCV introduction years included years before the country introduced PCV in its routine immunization program as well as the first year when PCV was introduced (taking into account the possible low vaccination coverage during the first year). Post–PCV introduction years included from the second year onward after PCV was routinely introduced.

### Ethical Considerations

Approval was granted by the ministries of health of the participating countries to report epidemiological and laboratory data to WHO as part of ongoing routine surveillance of vaccine-preventable diseases. This work was part of routine surveillance and did not require human subjects review.

## RESULTS

### Description of Suspected, Probable, and Confirmed Bacterial Meningitis Cases

From 2014 through 2019, 137 609 suspected meningitis cases from 58 countries were reported to GISN (Table 2). The number of sentinel sites that reported linked clinical and laboratory data varied by year: 110 sites in 54 countries (2014), 123 sites in 55 countries (2015), 126 sites in 58 countries (2016), 118 sites in 54 countries (2017), 123 sites in 51 countries (2018), and 105 sites in 45 countries (2019) reported data. Throughout the 6-year period of surveillance, most (n = 41 [70.7%]) countries reported during all years, whereas 17 countries reported between 2 and 5 years. Among the total of 149 sentinel sites that ever reported surveillance data during the 6-year period, 86 sentinel sites (57.7%) reported at least once each year for all 6 consecutive years. Also, 127 (85.2%) of the 149 total sentinel sites reported at least 10 months of surveillance data at any given year during the time period 2014–2019.

**Table 2.** Clinical, Laboratory, and Epidemiological Characteristics of Suspected, Probable, and Confirmed Meningitis Cases Detected and Reported to the World Health Organization Global Invasive Bacterial Vaccine-Preventable Disease Surveillance Network, 2014–2019

Characteristic	Suspected Meningitis Cases (n = 137 609)		Probable Bacterial Meningitis Cases (n = 11 798)		Confirmed Bacterial Meningitis Cases (n = 3576)a		Confirmed Sp Cases (n = 2177)		Confirmed Hi Cases (n = 633)		Confirmed Nm Cases (n = 766)	
	No. of Suspected Meningitis Cases	% of Suspected Meningitis (Column %)b	No. of Cases	% of Suspected Meningitis Cases (Row %)	No. of Cases	% of Probable Bacterial Meningitis Cases (Row %)	No. of Cases	% of Confirmed Bacterial Meningitis Cases (Row %)	No. of Cases	% of Confirmed Bacterial Meningitis Cases (Row %)	No. of Cases	% of Confirmed Bacterial Meningitis Cases (Row %)
Age group, mo												
0–5	52 452	(38.1)	5801	(11.1)	1334	(23.0)	864	(64.8)	252	(18.9)	218	(16.3)
6–11	25 421	(18.5)	1894	(7.5)	886	(46.8)	584	(65.9)	165	(18.6)	137	(15.5)
12–23	25 617	(18.6)	1459	(5.7)	502	(34.4)	277	(55.2)	88	(17.5)	137	(27.3)
24–59	34 119	(24.8)	2644	(7.7)	854	(32.3)	452	(52.9)	128	(15.0)	274	(32.1)
Mean age, mo	14		13		14		13		12		20	
Cases with age reported	137 609	…	11 798	(8.6)	3576	(30.3)	2177	(60.9)	633	(17.7)	766	(21.4)
Sex												
Male	81 330	(59.2)	6916	(8.5)	2084	(30.1)	1282	(61.5)	359	(17.2)	443	(21.3)
Cases with sex reported	137 304	…	11 766	(8.6)	3565	(30.3)	2171	(60.9)	630	(17.7)	764	(21.4)
Laboratory testing of CSF												
WBC count >100 cells/μL	5183	(6.9)	5183	(100.0)	1312	(25.3)	740	(56.4)	214	(16.3)	358	(27.3)
Cases with WBC counts analyzed	74 924	…	10 194	(13.6)	2660	(26.1)	1511	(56.8)	512	(19.2)	661	(24.8)
Protein >100 mg/dL	9441	(18.3)	4279	(45.3)	1069	(25.0)	693	(64.8)	139	(13.0)	237	(22.2)
Cases with protein analyzed	51 473	…	8100	(15.7)	1881	(23.2)	1115	(59.3)	303	(16.1)	463	(24.6)
Glucose <40 mg/dL	13 819	(24.6)	4155	(49.1)	1044	(55.7)	622	(56.8)	175	(58.1)	247	(51.6)
Cases with glucose analyzed	56 273	…	8468	(15.0)	1876	(22.2)	1096	(58.4)	301	(16.0)	479	(25.5)
CSF appearancec												
Clear	66 958	(76.6)	2634	(3.9)	1044	(39.6)	561	(53.7)	240	(23.0)	243	(23.3)
Turbid/cloudy	7258	(8.3)	7258	(100.0)	1691	(23.3)	1045	(61.8)	264	(15.6)	382	(22.6)
Cases with CSF appearance analyzed	87 431	…	11 470	(13.1)	3078	(26.8)	1830	(59.5)	557	(18.1)	691	(22.4)
Outcome												
Died	4010	(4.0)	675	(16.8)	277	(41.0)	189	(68.2)	24	(8.7)	64	(23.1)
Discharged alive with sequelae	1884	(1.9)	337	(17.9)	130	(38.6)	71	(54.6)	27	(20.8)	32	(24.6)
Discharged alive without sequelae	86 468	(86.3)	6739	(7.8)	1882	(27.9)	1127	(59.9)	309	(16.4)	446	(23.7)
Cases with outcome reported	100 217	…	8493	(8.5)	2525	(29.7)	1547	(61.3)	395	(15.6)	583	(23.1)
WHO Region												
African	61 386	(44.6)	5603	(9.1)	1833	(32.7)	1045	(57.0)	386	(21.1)	402	(21.9)
Americas	6614	(4.8)	1148	(17.4)	250	(21.8)	127	(50.8)	75	(30.0)	48	(19.2)
Eastern Mediterranean	18 714	(13.6)	815	(4.4)	229	(28.1)	197	(86.0)	25	(10.9)	7	(3.1)
European	2363	(1.7)	948	(40.1)	376	(39.7)	112	(29.8)	71	(18.9)	193	(51.3)
South-East Asia	34 167	(24.8)	1768	(5.2)	385	(21.8)	314	(81.6)	16	(4.2)	55	(14.3)
Western Pacific	14,365	(10.4)	1516	(10.6)	503	(33.2)	382	(75.9)	60	(11.9)	61	(12.1)
Total cases with region reported	137 609	…	11 798	(8.6)	3576	(30.3)	2177	(60.9)	633	(17.7)	766	(21.4)

Abbreviations: CSF, cerebrospinal fluid; Hi, *Haemophilus influenzae*; Nm, *Neisseria meningitidis*; Sp, *Streptococcus pneumoniae*; WBC, white blood cell; WHO, World Health Organization.

^a^The total of confirmed bacterial meningitis cases (n = 3576) is a subset of the probable bacterial meningitis cases. A total of 2130 confirmed cases met the probable bacterial meningitis cases definition, whereas 1446 confirmed cases did not meet the probable bacterial meningitis case definition but later had an etiology detected.

^b^The denominator for the indicated percentages was taken from the total cases reported/analyzed in each category as indicated in the column of number of suspected meningitis cases. (eg, 38.1% of cases were reported in the age group 0–5 months [52 452/137 609]).

^c^Indicators that contribute to classification of “probable bacterial meningitis.”

The largest contributor of suspected meningitis cases was in the 29 participating countries in the African Region (n = 61 386 [44.6%] of all suspected cases), followed by cases reported by the 3 countries in South-East Asia (n = 34 167 [24.8%]), the 4 countries in the Eastern Mediterranean (n = 18 714 [13.6%]), and 6 countries in the Western Pacific (n = 14 365 [10.4%]). The mean age of suspected meningitis cases was 14 months; 56.6% (n = 77 873) were reported among children aged <1 year. A slight majority (n = 81 330 [59.2%]) of cases were in males. Information on disease outcome was available for 100 217 children with suspected meningitis. Of these, 4010 (4.0%) died and 1884 (1.9%) were discharged but with sequelae (not specified).

**Figure 2.**
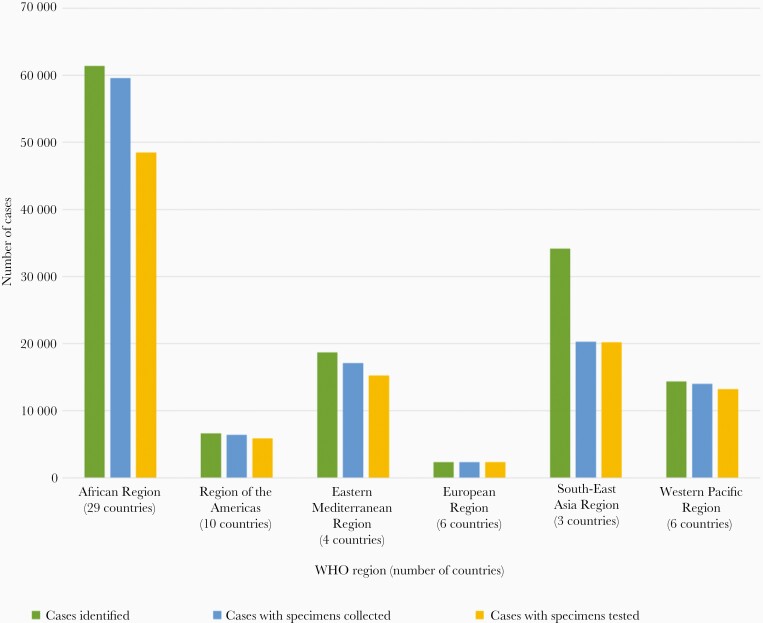
Number of children <5 years old with suspected meningitis who were reported and for whom specimens were collected and laboratory testing completed as part of the World Health Organization (WHO) Global Invasive Bacterial Vaccine-Preventable Disease Surveillance Network, by WHO region, 2014–2019. Across all regions from 2014 to 2019, 137 609 cases were identified from 58 countries, 119 808 specimens (87% of enrolled cases) were collected, and 105 418 specimens (88% of collected specimens) were tested by culture, polymerase chain reaction, and/or rapid diagnostic tests.

CSF specimens were collected from 119 808 (76.6%) suspected meningitis cases; 105 418 (88.0% of CSF specimens collected) were tested for presence of bacteria tested by culture and/or RDTs at the hospital laboratory before referral to the RRLs for PCR. The network used culture, RDT, and PCR for determining the positivity rates (microscopy not included). Specimens collected from all children with suspected meningitis included CSF and in some cases peripheral blood. CSF specimens were collected from 73.5% (n = 2628/3576) of the confirmed bacterial meningitis cases, blood specimens were collected from 2.0% (n = 71/3576), and both CSF and blood specimens were collected from 24.5% (n = 876/3576). (One case out of 3576 had a misreporting of having neither CSF or blood, but it was included as it was reported as a confirmed meningitis case that tested positive for Sp with a reported serotype.) The proportion of CSF specimens collected that were laboratory tested ranged by region, from 89.1% (n = 15 247/17 114) in the Eastern Mediterranean Region to 99.7% (n = 2344/2352) in the European Region (Figure 2). Among suspected cases, 8.6% (n = 11 798) were classified as probable bacterial meningitis. The proportions of suspected meningitis cases also reported as probable meningitis cases were largest for cases from the 6 countries in the European Region (40.1% of suspected meningitis cases, n = 948/2363) and from the 10 countries in the Americas (17.4%, n = 1148/6614), compared to 4.4% (n = 815/18 714) and 5.2% (n = 1768/34 167) of cases from the Eastern Mediterranean and South-East Asia regions, respectively.

Overall, a bacterial pathogen was identified in 3576 cases, confirming bacterial meningitis for 2.6% of suspected meningitis cases. Among these confirmed cases, 2130 cases (59.6%) met the probable meningitis case definition; the remaining confirmed cases that did not meet the probable meningitis case definition had a bacterial etiology detected by PCR or culture. The proportions of probable meningitis cases that were confirmed varied by WHO region, from 21.8% (Americas, n = 250) to 39.7% (European, n = 376). The predominant bacteria detected was Sp, which was identified in 2177 (60.9%) of the confirmed cases, followed by Nm, which was identified in 766 (21.4%) cases and Hi, identified in 633 (17.7%) cases (Table 2). The proportion of confirmed bacterial meningitis cases with each pathogen identified varied by WHO region. Whereas in the African Region, 57.0% of confirmed bacterial meningitis cases were due to Sp (n = 1045/1833), approximately one-third of confirmed bacterial meningitis cases in the European Region were due to Sp (n = 112 [29.8%]). In contrast, Sp comprised >75% of confirmed bacterial meningitis cases for 3 regions—Eastern Mediterranean (n = 197 [86.0%]), South-East Asia (n = 314 [81.6%]), and Western Pacific (n = 382 [75.9%]). The proportion of confirmed cases due to Hi ranged from 4.1% (n = 16/385) of cases in South-East Asia to 30.0% (n = 75/250) of confirmed bacterial meningitis cases reported by countries in the Americas. A greater range was reported for Nm, from 3.1% (n = 7/229) of confirmed cases due to Nm in the Eastern Mediterranean Region to 51.3% (n = 193/376) of cases due to Nm in the European Region.

Among all children aged <5 years with probable bacterial meningitis, 7.9% (n = 675) died and 4.0% (n = 337) were discharged alive but with sequelae. Among children with confirmed bacterial meningitis, 11.0% (n = 277) died and 5.1% (n = 130) were discharged from the hospital with sequelae (Table 2). Among the 277 children who died with confirmed bacterial meningitis, 189 (68.2%) had confirmed Sp. Compared with the case fatality ratio among children with Hi meningitis (24/395 [6.1%]), the case fatality ratio was greater among those infected by Sp (189/1547 [12.2%]; *P* < .001) and Nm (64/583 [11.0%]; *P* < .01).

### Pneumococcal Meningitis and Serotype Distribution

**Figure 3.**
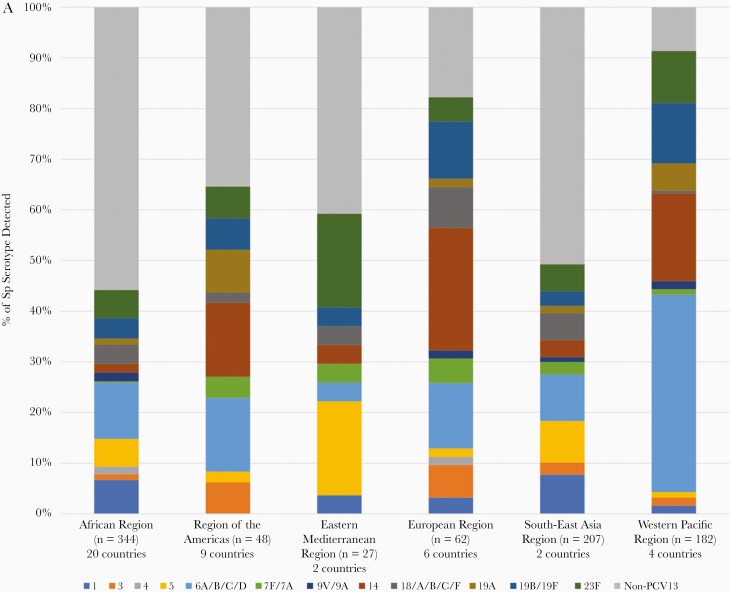

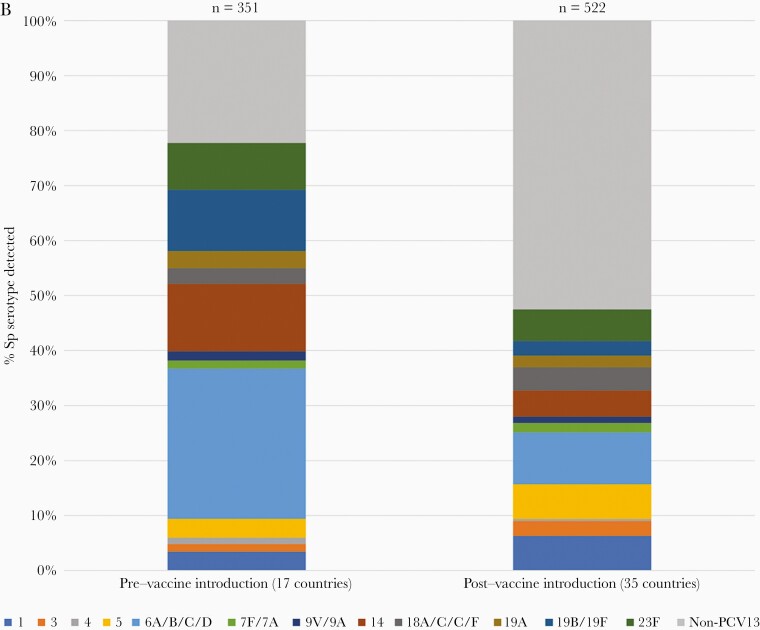
*A*, Serotype distribution of specimens that tested positive for *Streptococcus pneumoniae* (Sp) in the World Health Organization (WHO) Global Invasive Bacterial Vaccine-Preventable Disease (IB-VPD) Surveillance Network by WHO region, 2014–2019 (N = 873 specimens serotyped). N in brackets indicate the number of specimens that were serotyped for Sp in each WHO region. The number of countries with specimens serotyped is indicated below each WHO region. Countries include those that had and had not introduced PCV. Serotypes indicated in colors are those that are included in 13-valent pneumococcal conjugate vaccine (PCV13). *B*, Serotype distribution of specimens that tested positive for Sp in the WHO IB-VPD Surveillance Network by country PCV introduction status, 2014–2019 (N = 873 specimens serotyped). Pre–vaccine introduction includes years when a country had not yet introduced PCV nationally into its routine immunization program and the year of introduction, if applicable. Post–vaccine introduction includes full years when PCV was part of the country’s routine immunization program.

Pneumococcal serotype was determined for 873 of 2177 (40.1%) pneumococcal cases detected from the 6 WHO regions during 2014–2019 (Figure 3A). The distribution of serotypes for years in which countries that had not yet introduced PCV into their routine immunization programs was different from the serotype distribution for years after countries introduced PCV (Figure 3B; Pearson χ ^2^ = 143.10, *P* < .001). The most common PCV serotypes (and the percentage of vaccine-type serotypes reported) during post–PCV vaccination introduction years were the following: serotypes 6A/B/C/D (9.4%), serotype 1 (6.3%), and serotype 5 (6.3%). During post–PCV vaccine introduction years, 52.9% (n = 274) of the serotypes were non-PCV13 compared to 22.2% (n = 78) during pre–PCV vaccine introduction. There was a strong association between vaccine introduction status and whether the serotype was a vaccine type or not (Pearson χ ^2^ = 79.91; *P* < .001).

**Figure 4.**
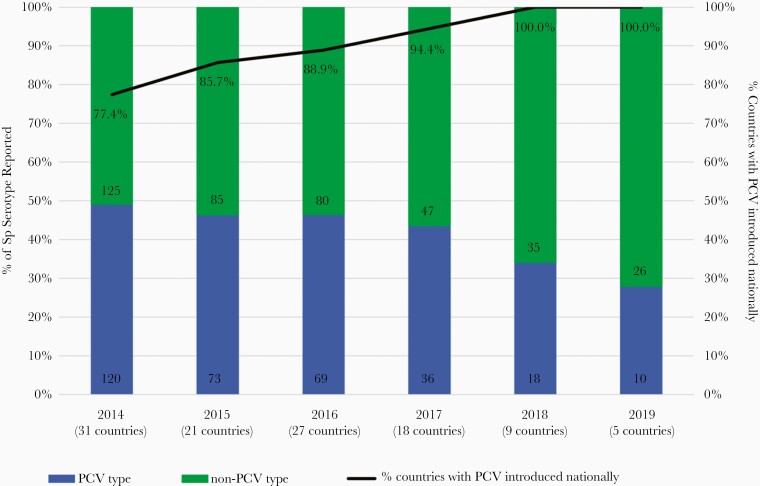
Serotype distribution of specimens that tested positive for *Streptococcus pneumoniae* (Sp) by year, 2014–2019, restricted to countries that introduced the 10- or 13-valent pneumococcal conjugate vaccine (PCV) during or prior to 2019. The proportion of countries included in this analysis that had introduced PCVs during or prior to each year is indicated by the line (and the percentage shown near the line). For example, in 2014, 24 of 31 countries (77.4%) had introduced PCV nationally during or prior to 2014. Among these 31 countries that reported serotype data in 2014, 120 (49.0%) cases had a PCV serotype whereas 125 (51.0%) had a non-PCV serotype. Although 7 countries reported in 2014 had not yet introduced PCV nationally, they were included in this analysis because they introduced the vaccine in the subsequent years (2015–2019). The numbers on the bars indicate the number of specimens with a detected Sp serotype including non-PCV type and PCV type. PCV10 serotypes include the following: 1, 4, 5, 6B, 7F, 9V, 14, 18C, 19F, and 23F; PCV13 includes the serotypes included in PCV10 plus 3, 6A, and 19A. The PCV type and non-PCV type were analyzed based on the PCV type that was introduced nationally by the respective countries during that particular year.

Among the countries that introduced PCV during or prior to 2019 and also reported pneumococcal serotype results, the proportion of infections caused by vaccine serotypes (serotypes in PCV10 or PCV13, depending on the vaccine in use in each country) declined over the 6-year period, from 49.0% in 2014 to 27.8% in 2019 (Figure 4); there was a significant decline in the proportion of infections caused by vaccine serotypes over time (χ ^2^ test for trend, *P* = .01).

### *H. influenzae* Meningitis and Serotype Distribution

All countries participating in GISN had introduced the Hib conjugate vaccine into their national immunization schedule prior to 2019. Among the 633 Hi detected from the participating countries in 2014–2019, 236 (37.3%) were serotyped; 82.6% (n = 195) were determined to be Hib. When comparing vaccine-type Hi (Hib) to nonvaccine type Hi (either serotypes a, b, e, f or categorized as non-Hib if the laboratory only serotypes for type b), Hib was detected by the majority across all WHO regions with a range of 57.9% (n = 11/19) in 2018 to 92.5% (n = 49/53) in 2016.

### Meningococcal Meningitis and Serogroup Distribution

Serogroup results were reported for 391 of the 766 (51.0%) confirmed bacterial meningitis cases in which Nm was identified. The most common serogroup was serogroup B, accounting for nearly half (n = 193 [49.4%]) of the specimens with a reported serogroup (Supplementary Figure 1). When comparing the proportion of Nm cases caused by serogroup B to the proportion caused by other serogroups (A, C, Y/W, and X), a significantly greater proportion of cases were due to serogroup B over time (χ ^2^ test for trend, *P* < .001; Supplementary Figure 2). Serogroup Y/W was the second most common, detected in 33.8% (n = 132), and serogroup C was detected in 15.1% (n = 59) of cases. The predominant serogroup detected varied by region. Serogroups Y/W were most common serogroups (87.6% of serogroups reported, n = 106) in the African Region whereas serogroup B was most common in the European Region (58.6%, n = 89), the South-East Asia Region (100%, n = 54) and Western Pacific Region (91.2%, n = 31) (Supplementary Figure 1). In the African Region, where epidemics of serogroup A were common prior to implementation of meningococcal serogroup A conjugate vaccine (MACV) vaccine, only 1 of 121 Nm cases (0.8%) that were serogrouped were type A.

### Results of External Quality Assessment

Up to 9 RRLs and 112 SSLs and NLs participated in the EQA exercise each year from 2014 to 2019. Based on a passing score of 75% for SSLs and NLs and 90% for RRLs, the proportion of laboratories that passed ranged from 83% to 92% during 2014–2019. Through capacity building and laboratory workshops coordinated by WHO, several participating NLs acquired PCR capacity from 2015 to 2019 as indicated by their reporting of results in serotyping/serogrouping using PCR on simulated CSF samples on the EQA exercise; in 2015, 8 of 16 laboratories (50.0%) passed the EQA whereas in 2019, 15 of 22 laboratories passed (68.2%).

## Discussion

The WHO-coordinated Global IB-VPD Surveillance Network captured case-based, laboratory-confirmed pediatric meningitis surveillance data from 58 countries representing all 6 WHO regions, providing a global landscape of this severe childhood infection. Pneumococcus was the most common pathogen detected from pediatric bacterial meningitis cases in GISN, despite increasing use of highly effective PCVs. The data also showed that among the children who died with confirmed bacterial meningitis, the majority had confirmed Sp infections. This highlights the importance of continuing meningitis surveillance with linked clinical and laboratory data. Not only will such surveillance be critical in detecting the number of pediatric meningitis cases, but it will also identify the type of pathogen as pneumococcus continues to be a fatal disease among children worldwide.

The continued occurrence of both vaccine-type and nonvaccine-type Sp meningitis cases suggests a role for both increased PCV coverage and higher-valent PCVs. There were regional differences in the bacterial etiologies and serotypes or serogroups most commonly causing meningitis. All countries in the surveillance network had introduced the Hib vaccine, but Hib continued to cause a modest portion of pediatric meningitis cases in all WHO regions even though the number of serotyped Hi samples was small. The lack of detection of meningococcal serogroup A in the African Region may reflect the varying detection of the disease across time. On another hand, it may show the impact of the MACV rollout in the routine immunization programs among the meningitis belt countries of sub-Saharan Africa that started from 2016 [3].

Bacterial meningitis diagnostics are often challenging, especially in laboratories in limited-resource settings, as the classical methods of culture rely heavily on highly perishable reagent components, such as sheep blood, which may not be reliably available year-round. The laboratory network is a key component of GISN. It was established to strengthen bacterial meningitis diagnosis globally, as 1 of the ultimate aims of the network is to reinforce national capacities for molecular diagnostics and transfer technologies from the global to regional to country level. As part of the objectives of GISN, WHO has taken the following initiatives to improve laboratory confirmation of invasive bacterial diseases caused by Sp, Hi, and Nm: supporting the sentinel and national laboratories to submit clinical specimens to RRLs to improve case detection and characterization; standardizing and validating laboratory procedures; linking laboratory and epidemiologic data for analysis; implementing and maintaining global quality assurance and quality control procedures; and conducting training resources to laboratory staff. The resources and expertise made available across all levels of laboratories have made the network unique, opening opportunities for network laboratories to seek support and feedback regarding surveillance methods and laboratory techniques. The laboratory network has a plan to continue efforts to improve laboratory confirmation of meningitis cases, including support for the development and implementation of sensitive and specific assays that are more accessible in resource-limited settings, such as direct PCR [6, 16].

During the ongoing coronavirus disease 2019 (COVID-19) pandemic and other public health emergencies of national or international concern, it is critical to have a well-functioning laboratory capacity to conduct surveillance, not only for vaccine-preventable diseases but also for emerging pathogens. Although a public health emergency can occur due to various pathogens, infrastructure and expertise built for conducting routine surveillance can be leveraged to rapidly characterize emerging outbreaks. In addition, the establishment of high-quality surveillance provides robust infrastructure to document trends during the emergence of other pathogens. For example, in 2020, the RRLs of GISN in South Africa and Brazil and Public Health England, which serves as the WHO Collaborating Center for pneumococcus, were still able to document significant and sustained reductions in Sp, Hi, and Nm invasive infections, due to the high-quality surveillance they have maintained despite the perturbations caused by the COVID-19 pandemic [17].

Since the start of GISN, many participating laboratories were able to expand their surveillance systems beyond detecting and characterizing invasive bacterial diseases. For example, 1 of the reference laboratories in Bangladesh has successfully utilized their bacterial capacities to conduct typhoid surveillance in addition to invasive bacterial diseases [18]. Sentinel sites of GISN in Ghana, India, and Uganda also conducted pilot surveillance for typhoid and paratyphoid fever from 2016 to 2019. Additionally, RRLs, NLs, and SSLs of GISN have leveraged their bacterial capacities to detect and characterize diphtheria, by culture and PCR, after participating in WHO laboratory workshops on invasive bacterial diseases and diphtheria that were held in the African, European, Eastern Mediterranean, South-East Asia, and Western Pacific regions in 2017 and 2018. Integrating diphtheria diagnostics into laboratories during the 2017 diphtheria outbreak in Bangladesh was particularly effective as part of outbreak response because these laboratories were already well-established as part of GISN. From 2020, many of the laboratories and staff of GISN was repurposed to test for severe acute respiratory syndrome coronavirus 2 in their own countries. These examples demonstrate how the strengths of GISN were utilized and expanded beyond routine surveillance for invasive bacterial vaccine-preventable diseases for which the network was originally designed.

This analysis is subject to several limitations. The overall diagnostic yield and bacterial isolation rate was generally low in the network due to limited bacteriology capacity in many of the participating countries and widespread antibiotic use before initial laboratory testing. Molecular diagnostics such as serotyping capacity for pneumococcus was also limited since less than half (873/2177 [40%]) of all confirmed Sp cases were serotyped globally. This was often due to limited laboratory resources and funding available for SSLs and NLs to ship the specimens for further testing at RRLs. Competing priorities at country and regional levels led to challenges in building and sustaining national laboratory capacities as well as maintaining surveillance capacities at the sentinel hospital sites. Since GISN primarily enrolled hospitalized pediatric meningitis cases through sentinel site surveillance, this analysis may underestimate the full burden of disease at the country level. This can be due to not having all cases presenting at the sentinel sites from lack of access to a healthcare facility or the cases presenting at different hospitals or clinics that were not conducting surveillance through GISN. However, among the sentinel sites that participated in GISN from 2014 to 2019 globally, specimens were collected and laboratory testing was completed for >85% of all identified cases. This shows that the vast majority of the cases identified in GISN had clinical specimens collected for laboratory testing. It is possible that some sentinel hospitals preferentially submitted information for suspected meningitis cases with collected CSF specimens. However, regular site assessments were conducted at sentinel hospitals and laboratories to evaluate their performance in conducting surveillance. Recommendations were frequently provided to implement corrective actions in case the sites were deviating from standard protocol. Having both clinical and laboratory data linked in a global surveillance network has been an advantage when understanding the prevalence of the disease as well as their characteristics, such as the frequency of serotypes/serogroups of Sp, Hi, and Nm.

During the beginning of GISN, most of the participating countries financed their surveillance networks with the support of Gavi, the Vaccine Alliance. As these countries phased out of eligibility for Gavi funding, the number of countries reporting surveillance data to GISN decreased. The highest number of sentinel sites participating in GISN that reported data was 2016 when 126 sites in 58 countries reported data to GISN, but this dropped to 105 sites in 45 countries in 2019. Concurrently, the number of countries contributing Sp serotype results decreased over time as countries either discontinued meningitis surveillance or stopped reporting serotype results. Although the number of countries reporting serotype results decreased, however, this is not expected to bias the estimate of PCV impact—the decrease in the proportion of pneumococcal infections caused by vaccine serotypes over time. Additional challenges included appropriate timing and conditions for specimen transport and sufficient availability of supplies and test kits. While the network includes data from a wide range of countries globally, the data collected by GISN are not necessarily representative of all invasive bacterial diseases globally, within regions or within countries. Since this network primarily focused on reporting the 3 bacterial pathogens, Sp, Hi, and Nm, this analysis may underreport pediatric meningitis cases that are caused by other bacterial pathogens such as group B *Streptococcus*, as these may had been detected yet unreported after testing positive by culture.

Additionally, laboratories participating in the annual EQA exercise faced logistical challenges and restrictions in shipping or delivery of infectious materials, accounting for a decline in the number of laboratories reporting EQA results in some years. Finally, the cell count, glucose, and protein level testing that was used to classify cases into probable bacterial meningitis was mostly performed at pathology or biochemistry laboratories at the sentinel hospital level, and these methodologies were not part of the EQA activities. Therefore, the quality of the data to classify the probable bacterial meningitis cases in GISN cannot be verified in some cases.

Despite the challenges in building national bacteriology capacity and regional and global surveillance networks, GISN successfully monitored the global burden and etiology of IB-VPD in a widely geographically representative set of primarily LMICs. The network has also supported national laboratories to hone their molecular capacities, opening doors to detect other viral and bacterial diseases including emerging pathogens. Quality control management exercises have encouraged all levels of laboratories to maintain and improve their capacities. From the start of the network, the sharing of the surveillance data across countries and regions has improved the quality of surveillance data and its use—from understanding the disease burden by monitoring trends to assessing vaccine impact. The WHO African Region, for example, has utilized this network to investigate the disease burden of invasive bacterial diseases [19]. Some countries, such as The Gambia and Bangladesh, were successful in informing national vaccine policy through the usage of surveillance data from GISN [20, 21].

Maintaining case-based, active surveillance with laboratory confirmation for bacterial meningitis and other prioritized vaccine preventable diseases is a critical part of the global agenda, as highlighted in the Immunization Agenda 2030 and the Defeating Meningitis roadmap [7, 22, 23]. The results from this analysis of GISN have demonstrated the critical value of a globally coordinated network to understand and monitor invasive bacterial diseases through surveillance data. Thus, the prioritization of ministries of health allocating funding for surveillance is vital in maintaining their capacities to conduct high-data-quality surveillance on prioritized vaccine-preventable diseases that remain a concern in some countries. In conclusion, the systematic approach taken by the WHO-coordinated Global IB-VPD Surveillance Network, the geographical reach, and quality management implemented at all levels of laboratories have allowed this network to successfully investigate the regional and global disease patterns of pediatric bacterial meningitis.

## Supplementary Data

Supplementary materials are available at *The Journal of Infectious Diseases* online. Consisting of data provided by the authors to benefit the reader, the posted materials are not copyedited and are the sole responsibility of the authors, so questions or comments should be addressed to the corresponding author.

jiab217_suppl_Supplementary_Data

## Notes

***Acknowledgments.*** The authors thank the hard work and dedication of all the partners including laboratorians, clinical staff, surveillance officers, and data managers who contribute to ongoing surveillance at each site that participates in the World Health Organization–coordinated Global IB-VPD Surveillance Network.

***Financial support.*** The WHO-coordinated Global IB-VPD Surveillance Network was supported through the Gavi Alliance’s Data Strategic Focus Area portion of the Partners’ Engagement Framework.

Supplement sponsorship. Supplement sponsorship was from the US Centers for Disease Control and Prevention, grant number U2GGH002145-02-00.

***Potential conflicts of interest.*** All authors: No reported conflicts of interest. All authors have submitted the ICMJE Form for Disclosure of Potential Conflicts of Interest. Conflicts that the editors consider relevant to the content of the manuscript have been disclosed.
