# Supplementary material for: The Global Landscape of Pediatric Bacterial Meningitis Data Reported to the World Health Organization–Coordinated Invasive Bacterial Vaccine-Preventable Disease Surveillance Network, 2014–2019
Source: J Infect Dis. 2021 Sep 1;224(Suppl 3):S161–73. doi: 10.1093/infdis/jiab217 (PMC8409679; doi:10.1093/infdis/jiab217)
Supplement: jiab217_suppl_Supplementary_Data [file jiab217_suppl_supplementary_data.docx]

**Supplemental Figure 1.** Serogroup distribution of specimens which tested positive for *Neisseria meningitidis* by WHO Region, including all countries regardless of meningococcal conjugate vaccine introduction, 2014-2019 (N=391 specimens serogrouped). The number of countries that reported these specimens are indicated below per WHO Region.^[[1]](#footnote-1)^


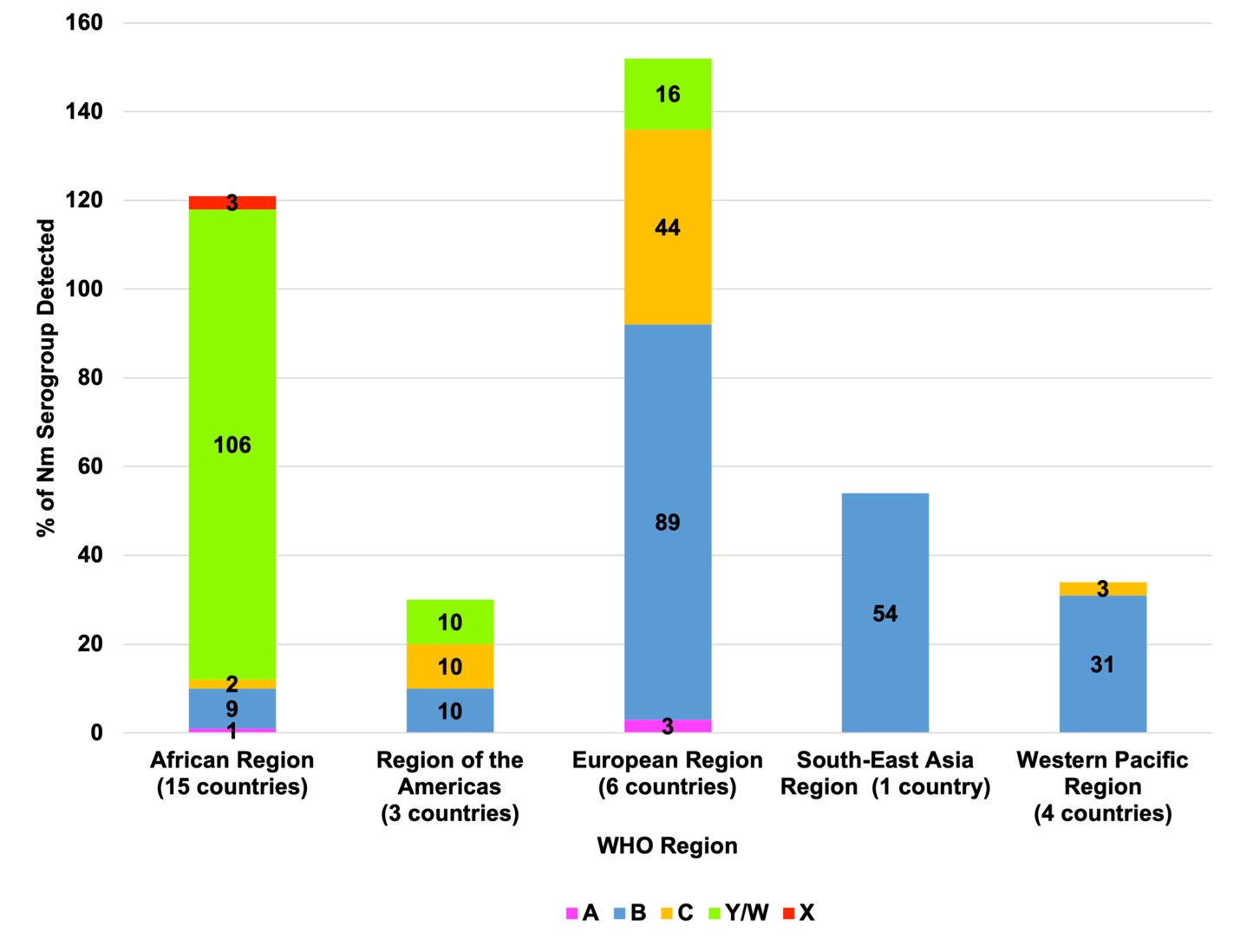


**Supplemental Figure 2.** Serogroup distribution of specimens which tested positive for *Neisseria meningitidis* by year from 2014-2019 (N=391 specimens serogrouped) including all countries regardless of meningococcal conjugate vaccine introduction.


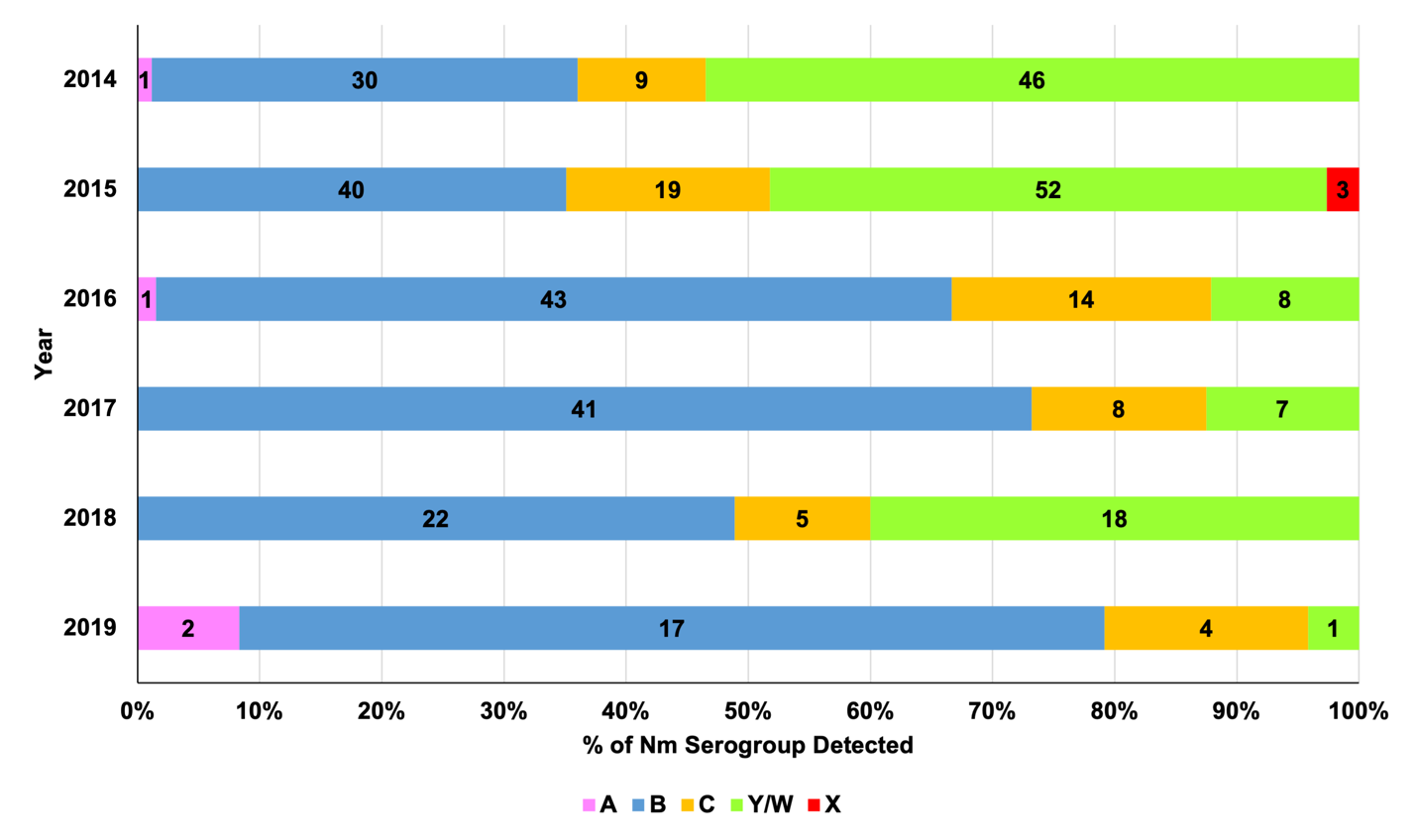


1. Countries from the Eastern Mediterranean Region were not included as no Nm serogroup results were reported during 2014-2019. [↑](#footnote-ref-1)
